# Women and evaluation of inequalities in the distribution of risk factors for Chronic non-communicable diseases (NCD), Vigitel 2016–2017

**DOI:** 10.1590/1980-549720200058

**Published:** 2020-06-05

**Authors:** Deborah Carvalho Malta, Regina Tomie Ivata Bernal, Quéren Hapuque de Carvalho, Jill P. Pell, Ruth Dundas, Alastair Leyland, Leda Lúcia Couto de Vasconcelos, Lais Santos de Magalhaes Cardoso, Sheila Rizzato Stopa, Mauricio Lima Barreto

**Affiliations:** I Nursing School, Universidade Federal de Minas Gerais – Belo Horizonte (MG), Brazil; II Institute of Health and Wellbeing, University of Glasgow, Lilybank Gardens – Glasgow, United Kingdom; III MRC/SCO Social and Public Health Sciences Unit, University of Glasgow – Glasgow, Reino Unido; IV Department of Medicine, Universidade Federal de Sergipe – Lagarto (SE), Brazil; V Secretary of Health Surveillance, Ministério da Saúde – Brasília (DF), Brazil; VI Centro of Health Data and Knowledge Integration, Fundação Oswaldo Cruz – Salvador (BA), Brazil

**Keywords:** Socioeconomic factors, Noncommunicable Diseases, Health Surveys, Health Evaluation, Cash Transfer, Social Programmes, Iniquidades, Doenças Crônicas não Transmissíveis, Inquérito sobre Saúde, Avaliação em Saúde, Programas Sociais

## Abstract

**Objective:**

To compare the distribution of chronic non-communicable diseases (CNCD) indicators among adult female beneficiaries and non-beneficiaries of the Bolsa Família Program (BFP) in Brazilian capitals.

**Methods:**

Analysis of Vigitel telephone survey data in 2016 and 2017. Gross and adjusted prevalence ratios (PR) and their respective confidence intervals were estimated using Poisson Regression model.

**Results:**

Women with BF have lower schooling, are young people, live more frequently in the Northeast and North of the country. Higher prevalence of risk factors were found in woman receiving BF. The adjusted PR of the BF women were: smokers (PR = 1.98), overweight (PR = 1.21), obesity (PR = 1.63), fruits and vegetables (PR = 0.63), consumption of soft drinks (PR = 1.68), bean consumption (PR = 1.25), physical activity at leisure (PR = 0.65), physical activity at home (PR = 1.35), time watching TV (PR = 1.37), self-assessment of poor health status (PR = 2.04), mammography (PR = 0.86), Pap smears (PR = 0.91), hypertension (PR = 1.46) and diabetes (PR = 1,66). When women were compared among strata of the same schooling, these differences were reduced.

**Conclusion:**

Worst indicators among women receiving BF reflect social inequalities inherent in this most vulnerable group. The study also shows that BF is being targeted at the most vulnerable women.

## RESUMO

**Objetivo:**

Comparar a distribuição de indicadores de doenças crônicas não transmissíveis (DCNT) entre mulheres adultas beneficiárias e não beneficiárias do Programa Bolsa Família (PBF) nas capitais brasileiras.

**Métodos:**

Análise de dados do Sistema de Vigilância de Fatores de Risco e Proteção para Doenças Crônicas por Inquérito Telefônico (Vigitel) em 2016 e 2017. Foram estimados as razões de prevalência (RP) brutas e ajustadas e seus respectivos intervalos de confiança usando o modelo de regressão de Poisson.

**Resultados:**

Mulheres do PBF tem menor escolaridade, são mais jovens e vivem com maior frequência nas regiões Nordeste e Norte do país. Prevalências mais elevadas de fatores de risco foram encontradas nas mulheres beneficiárias do PBF. A RP ajustada por idade das mulheres com BF foram: fumantes (RP = 1,98), excesso de peso (RP = 1,21), obesidade (RP = 1,63), frutas e hortaliças (RP = 0,63), consumo de refrigerantes (RP = 1,68), consumo de feijão (RP = 1,25), prática de atividade física no lazer (RP = 0,65), atividade física no domicílio (RP = 1,35), tempo assistindo à TV (RP = 1,37), autoavaliação do estado de saúde ruim (RP = 2,04), mamografia (RP = 0,86), Papanicolau (RP = 0,91), hipertensão (RP = 1,46) e diabetes (RP = 1,66). Quando comparadas as mulheres entre estratos de mesma escolaridade, as diferenças entre os fatores de risco foram reduzidas.

**Conclusão:**

Piores indicadores entre mulheres que recebem BF refletem desigualdades sociais inerentes a esse grupo mais vulnerável. O estudo evidencia também que o PBF está sendo destinado às mulheres mais vulneráveis.

## Introduction

Chronic non-communicable diseases (CNCD) are considered the main causes of death and disability in the world population, responsible for high costs and financial burdens on individuals, societies and health systems^1,2^. Studies show that CNCDs affect quality of life and well-being^3,4^, as well as the importance of social determinants, particularly poverty, in the occurrence of CNCDs, with the worst indicators in the most vulnerable and socially marginalized population^5–7^. In addition, health inequities cause unequal possibilities related to the scientific and technological advances that have occurred in this area, changing the chances of exposure to the factors that determine health and illness, further expanding inequalities and the risks of illness and death^7^.

In this regard, several social protection strategies have been used in the world to promote social mobility and face the problem of hunger and misery. Among them, conditional cash transfer (CCTs) programs have stood out for their contractual design and governance structure, which promotes the individual’s positive behavior, as a central element, and not care ^8^.

In the Brazilian context, *the Bolsa Família* Program (BFP) was created by the federal government through Provisional Measure No. 132^9,10^. The program is a social welfare action, being one of the largest direct cash transfer programs in the world and, by legal definition, women are the preferred holders for receiving the benefit (Law No. 10,836/2004)^11,12^.

Since 2006, the Surveillance System for Risk and Protection Factors for Chronic Diseases by Telephone Survey (Vigitel) has been monitoring indicators of these diseases through telephone surveys. In 2016, a specific question was added about receiving the BFP benefit in order to monitor vulnerable populations in relation to CNCD indicators, especially women, who make up more than 90% of the beneficiary population^11,12^.

Thus, this is the first study to analyze national data on CNCD risk factors among women beneficiaries of the BFP by telephone survey. It is noteworthy that this is a gap in the national literature, given the vulnerability of this public and the evidence that CNCDs and their risk factors (RF) are more frequent in the low-income population^6,13^.

The aim of the present study was to compare the distribution of CNCD indicators among adult women beneficiaries and non-beneficiaries of the BFP in Brazilian capitals and the Federal District.

## Methods

This is a population-based epidemiological study with a cross-sectional design, with data from Vigitel, years 2016 and 2017, from the 26 Brazilian capitals and the Federal District. The surveyed population was comprised of adult women (≥ 18 years and over). Approximately two thousand interviews are carried out per capital, about 54 thousand interviews each year^14.^ In the current study, 3,330 women benefiting from the PBF and 63,152 non-beneficiaries were studied. Data on the sample design can be read in other publications^14,15^.

In 2016, the following question was introduced: “Do you or someone in your family who lives at home receive Bolsa Família (BF)?”. This is the first study to analyze this question. Currently, 92% of women are the beneficiary of the family, regardless of the type of family arrangement to which they belong^12^. Thus, indicators from the Vigitel database for adult women were analyzed.

The prevalences were compared, with the respective 95% confidence intervals (95% CI) of the following indicators:

- Risk factors:
  a) smokers (reporting smoking, regardless of quantity);
  b) body weight (overweight — body mass index ≥ 25 kg/m^2^; obesity — body mass index ≥ 30 kg/m^2^) - the absent values of overweight and obesity suffered imputation, according to the methodology used by Vigitel and previously described^14;^
  c) regular consumption of soft drinks or artificial juice (five or more days a week);
  d) habit of watching TV for 3 hours or more a day;
  e) abusive consumption of alcoholic beverages (four or more doses for women) on the same occasion in the last 30 days;
  f) self-assessment of poor health status; g) referred morbidities (previous medical diagnosis of hypertension and diabetes);
- Protective factors:
  h) recommended consumption of fruits and vegetables (five or more servings daily, five or more days a week);
  i) regular consumption of beans (five or more days a week);
  j) physical activity (PA) in free time (PA ≥ 150 minutes in the free time domains);
  k) conducting tests for early detection of cancer in women (mammography for women aged 50 to 69 years, in the last two years; and Pap smear for women aged 25 to 59 years, in the last three years).

For this analysis, it was necessary to join the Vigitel 2016 and 2017 databases and select only the women who received or did not receive the BFP benefit. The choice to study two years was a methodological option to increase the sample size of women who received the benefit.

It was necessary to calculate post-stratification weights, since the sample of adults interviewed by Vigitel was extracted from the register of residential telephone lines existing in each city, which only allows, strictly, inferences for the adult population residing in households covered by fixed telephone network. Thus, the use of post-stratification weights aims to minimize the addiction resulting from the low coverage of landline registrations, especially in the North and Northeast regions^14,15^.

In order to construct the post-stratification weights of this subsample of women, the estimate of the female population with or without PBF obtained by the 2016 National Household Sample Survey (PNAD) was used as the reference population^16^. The variables age, education and region were used in the construction of the weights, as these characteristics are associated with the possession of the PBF. Weights were obtained using the rake method^15^.

The descriptive analyzes consisted of calculating the distribution of the proportions of women who receive and do not receive the benefit of the PBF, according to sociodemographic characteristics and NCD indicators. The prevalence and the prevalence ratio of the indicators were analyzed among the population with and without PBF, using the Poisson regression model with robust variance, with adjustment for age. A stratified analysis was also carried out according to the different schooling strata (zero to eight years, nine to 11 years and 12 years and more), comparing women with the same level of education, beneficiaries or not of the PBF, with adjustment for age. Data analysis was performed using the Statistical Software for Professional (Stata) statistical program, using the commands of the survey module, taking into account the weights and, therefore, the sample’s representativeness.

The Vigitel survey was approved by the National Commission for Ethics in Research for Human Beings, of the Ministry of Health, opinions 13.081/2008 and 355.590/2013. The signature of the Free and Informed Consent Form (ICF) in this survey was replaced by the verbal consent of the interviewee at the time of the telephone call.

## Results

Among the 66,482 women studied in 2016 and 2017, 3,330 live in households that received BF during the study period, and these residents are concentrated mainly in the Northeast and North regions — 1,674 (50.3%) and 922 (27.7%), respectively. There is also a greater proportion of these beneficiaries between the ages of 25 to 54 years old (2,046/61.4%), without education and/or with incomplete elementary school (926/27.8%) and complete high school and or incomplete higher education (1,538/46.19%) (Table 1).

The distribution of CNCD risk and protection factors for women with and without BFP, in the group of 26 capitals, and the estimated prevalence ratios (PR) are shown in Table 2. In general, higher prevalence of risk factors and lower frequencies of protective factors were found in women benefiting from the BFP.

The age-adjusted prevalence ratio (PR) for female smokers was 1.98 (95% CI 1.61 – 2.43). Overweight and obesity were higher among women with PBF: PR = 1.21 and 1.63 (95% CI 1.14 – 1.29; 1.44 – 1.85), respectively. Regarding eating habits, the recommended consumption of fruits and vegetables was lower among women with GMP (PR = 0.63; 95% CI 0.54 – 0.73), but there was a higher consumption of beans (PR = 1.25; 95% CI 1.18 – 1.32) and soft drinks (PR = 1.68; 95% CI 1.43 – 1.98). Women benefiting from PBF had less practice of PA during leisure (PR = 0.65; 95% CI 0.57 – 0.75); higher PA at home (PR = 1.35; 95% CI 1.29 – 1.41); longer time watching TV (PR = 1.37; 95% CI 1.21 – 1.55); worse self-assessment of health status (PR = 2.04; 95% CI 1.56 – 2.66); lower coverage of mammography (PR = 0.86; 95% CI 0.79 – 0.94) and Pap smear (PR = 0.91; 95% CI 0.87 – 0.95); and higher prevalence of self-reported morbidities, such as hypertension and diabetes (PR = 1.46 and 1.66; 95% CI 1.29 – 1.64; 1.29 - –2.07, respectively). There were no statistically significant differences between the prevalence of beneficiaries and non-beneficiaries for alcohol abuse (Table 2).

Table 3 shows the prevalence, PR and age-adjusted PR of CNCD risk and protection factors among women with and without BFP from the same age-adjusted level of education (zero to eight years of study). Women who are beneficiaries of the BFP with low education have a higher prevalence of tobacco use (PR = 1.51; 95% CI 1.16 – 1.97), a higher prevalence of obesity (PR = 1.24; 95% CI 1.06 – 1.45), lower recommended consumption of fruits and vegetables (PR = 0.71; 95% CI 0.56 – 0.89), less PA practice at leisure (PR = 0.79; 95% CI 0.63 – 0, 98) and worse self-assessment of health status (PR = 1.46; 95% CI 1.07 – 1.97). As a protection indicator, there was a higher consumption of beans (PR = 1.09; 95% CI 1.01 – 1.17).

In Table 4, women with and without BFP with average schooling (nine to 11 years of study) are compared, adjusted for age. The adjusted PR shows that women benefiting from the BFP have worse indicators, such as: smoking (PR = 1.54; 95% CI 1.14 – 2.09), overweight (PR = 1.11; 95% CI 1.02 – 1.21), obesity (PR = 1.29; 95% CI 1.08 – 1.54), lower recommended consumption of fruits and vegetables (PR = 0.81; 95% CI 0.67 – 0.97), less practice of leisure-time physical activity (PR = 0.82; 95% CI 0.71 – 0.95), greater PA at home (PR = 1.18; 95% CI 1.11 – 1.25), state self-assessment poor health (PR = 1.69; 95% CI 1.16 – 2.44) and higher self-reported prevalence of diabetes (PR = 1.60; 95% CI 1.22 – 2.10). As a protective factor, greater consumption of beans was observed (PR = 1.13; 95% CI 1.06 – 1.21).

Table 5 contains the prevalence and PR of the risk and protective factors for CNCDs among women with and without BFP with schooling of 12 years or more, adjusted for age. Among women beneficiaries of the BFP with a high level of education, the differences were reduced, but worse performance was still found for overweight (PR = 1.27; 95% CI 1.01 – 1.61), soft drink consumption (PR = 2.01; 95% CI 1.30 – 3.12) and self-reported hypertension (PR = 1.67; 95% CI 1.07 – 2.60). The other variables did not show significant differences.

## Discussion

This is the first study that analyzes data from Vigitel for adult women who are beneficiaries of the BFP. This population is more vulnerable and has a lower family income. Among the sociodemographic indicators, the study highlighted: less education, greater concentration in the Northeast Region and younger population, among women beneficiaries of BFP. Among the indicators analyzed here, in general, the prevalence of risk factors was higher among women who receive the BFP benefit. They have a higher prevalence of smoking, overweight and obesity, less consumption of fruits and vegetables, higher consumption of soft drinks, however, on the other hand, the consumption of beans was higher. These women also had less PA practice during leisure time, however greater PA at home, or performing heavy cleaning tasks at home, in addition to more time watching TV. They had a worse self-assessment of their health status considered poor, less coverage of mammography and Pap smears and higher prevalence of self-reported morbidities (hypertension and diabetes). The study points out worse indicators in the poorest populations.

These differences decreased when women between strata with the same education were compared. When comparing the women beneficiaries of the PBF with 12 years or more of study with the others, the prevalence of risk factors was reduced, and the differences found were: higher prevalence of overweight, higher consumption of soft drinks and higher prevalence of hypertension self-reported among women with BFP. All other variables were similar, revealing that education is a major protective factor for women’s health.

These data highlight the importance of social determinants, particularly poverty, in the occurrence of CNCDs, with the worst indicators in the most vulnerable population^5,17^.

The prevalence of smoking indicators was higher among women beneficiaries of BFP. Studies indicate that populations with higher education, income and better socioeconomic conditions have lower prevalence of tobacco use^18^, which has been explained by the greater access to information on the harms of this habit. It is noteworthy that the differences disappeared among more educated women, showing that their disadvantage can be overcome through greater investment in education.

Foods such as fruits and vegetables are considered protective and prevent cardiovascular diseases and cancers^4,19^. Vigitel’s recommended consumption of fruits and vegetables (FLV) is a proxy for what would be recommended by the World Health Organization, or the daily consumption of 400g/day of fruits and vegetables^14,19,20^.

Likewise, beans are a protective food, as they are rich in fiber and nutrients, resulting in greater satiety and obesity prevention^21^, and are recommended by the Food Guide for the Brazilian Population, which recognizes its role in the national culinary tradition^22^. Because it is a lower cost food and has an important participation in traditional Brazilian cuisine, its consumption is higher among lower income populations. On the contrary, soft drinks, fats, sugars and salt are foods that increase the risk of CNCD^4,20,22^.

The study pointed out that women from the BFP have less consumption of FLV and more of soft drinks; the exception was beans. The Strategic Action Plan to Combat CNCDs encourages countries to adopt measures to regulate ultra-processed foods with a high sugar, salt and fat content, as well as measures that can increase production and consumption and reduce prices of fresh food such as vegetables^4,22^. These measures would bring great benefits to the population^4,22^, especially to the poorest, such as women beneficiaries of the BFP and their families. It is also noteworthy that having more education brought important benefits to the diet of the women studied here, regardless of the economic level.

Leisure-time PA was less practiced by women benefiting from the PBF, in contrast to domestic PA, represented by heavy cleaning, as well as longer TV time or sedentary leisure. Leisure time PA is associated with populations with high education and income, due to greater access to spaces for PA practice and greater knowledge about the benefits of PA^23^. On the contrary, PA practices during work and commuting to work are associated with low-income populations. In a special way, PA in the home is more affectionate to women, due to a macho aspect of Brazilian society, which considers that domestic activities should be performed by women^24^. It was also found that in the analysis stratified by education, these differences were reduced, pointing out that it is possible to change this reality, investing in public policies of access to public spaces for PA practice, as well as improving the population’s income and education, aiming to decrease these inequalities^5^. Data from the National Health Survey indicate that overweight affects more than half of women, and obesity, 24%, revealing the extent of the problem in the country^25^. Also, obesity can affect the achievement of the global goal of reducing CNCD mortality^26^. The study shows that, although it is a widespread problem, it is even more serious among women beneficiaries of PBF, especially with low education.

Studies indicate that the prevalence of at least one chronic disease increased with age and was higher among women^27^, in addition to the presence of multimorbidities (two diseases and three or more diseases)^28,29^.

Self-assessment of poor health status was more frequent among women beneficiaries of BFP, but, when stratified by schooling, in strata with 12 or more years of study, this difference disappeared, which is in accordance with the literature^29^. This indicator is classically associated with worse health and life conditions, low income and the elderly population, and is an important predictor of mortality^30,31^.

The study showed that hypertension and diabetes morbidities were more prevalent among women with BFP, or low income, which has been confirmed in other studies, both by Vigitel, pointing out that the prevalence of hypertension in the population with low education was three times as high^32^ than for diabetes^33^, with frequencies up to four times higher in the population with low education. Also, in international studies^35^, an association was found between educational level and diabetes mellitus, after adjusting the variables income and occupation^34^. The explanations for this finding are schooling, as a proxy for socioeconomic level, and less access to health promotion practices, such as healthy eating, PA, access to medicines and health services^17,35^.

The coverage of preventive cancer exams was lower among women beneficiaries of PBF, with frequencies below that recommended by the Strategic Action Plan for Coping with Chronic Diseases in Brazil, which foresees reaching 75% of mammography coverage and 85% of Pap smears in 2022^36^. It is noteworthy that the prevalence of oncotic cytology (Pap smear) is higher, possibly because they are performed by the Family Health Strategy, through the Unified Health System (SUS) ^35^.

Alcohol abuse did not differ between the two groups of women, but, when stratified for 12 years, it was slightly higher among women with higher income, which is consistent with other studies^14^.

The study highlights health inequities, which are the worst indicators in the population benefiting from BFP, revealing the importance of social determinants in the health-disease process. Higher income populations have easier access to health services and promotion practices^4,5^. These results reflect the importance of investments in improving living conditions and education, which can directly interfere with health indicators.

Cash transfer programs are fundamental in tackling inequalities, especially with regard to strengthening actions related to gender, in the production of equity^37^. The study showed greater vulnerability of this population, which should also be prioritized by health services, in positive discrimination with regard to access to services, educational practices and public policies for health promotion. Thus, this study may contribute to the definition of health policies aimed at the prevention and control of these diseases.

Among the limits of the study, the use of telephone interviews stands out, seeking to reduce this bias using post-stratification weights. The fact that the information is self-reported can also result in information bias, although national and international experience highlights that variables such as arterial hypertension and health status assessment can obtain good estimates using this methodology, in addition to presenting advantages such as speed of information, sensitivity and low cost^38^. Due to the reduced number of interviews with BFP, it was decided to analyze 2016 and 2017, reducing the standard error of the estimates. Another limitation is the fact that the respondent is not the beneficiary of the program, since the question is about the benefit in the family. In this case, a *proxy* of the results is made. In addition, the cross-sectional design of the study does not allow the establishment of a temporal cause and effect relationship.

## Conclusion

The study showed differences in risk and protection factors among women, with worse indicators among those who receive the BFP benefit. It is noteworthy that this does not reflect a causal relationship between receiving BFP and having a worse performance, but rather the social inequalities inherent to this most vulnerable group, characterized by little access to healthy food, places to practice PA, health services and practices health promotion, in addition to differences in schooling and less information about disease prevention and health promotion practices.

The study also shows that the BFP is being targeted at women with the worst indicators, demonstrating the importance of this program, which works on social determinants of health and the reduction of inequalities, and the use of Vigitel in this assessment.

## Figures and Tables

**Table 1:** Frequency of the sample of women aged 18 or over by age, education level and region according to the *Bolsa Família* Program. Set of capitals and Federal District. Surveillance System for Risk and Protection Factors for Chronic Diseases by Telephone Survey (Vigitel), Brazil. 2016 and 2017.

Variables	Has Bolsa Família		No Bolsa Família		Total	
	n	%	n	%	n	%
Age (years)						
18 to 24	420	12.61	4,516	7.15	4,936	7.42
25 to 34	654	19.64	6,683	10.58	7,337	11.04
35 to 44	796	23.90	8,606	13.63	9,402	14.14
45 to 54	596	17.90	10,706	16.95	11,302	17.00
55 to 64	486	14.59	12,867	20.37	13,353	20.09
≥ 65	378	11.35	19,774	31.31	20,152	30.31
Schooling level						
None/incomplete primary	926	27.81	13,408	21.23	14,334	21.56
Complete primary/incomplete high school	626	18.80	6,811	10.79	7,437	11.19
Complete high school/incomplete third level	1,538	46.19	23,253	36.82	24,791	37.29
Complete third level	240	7.21	19,680	31.16	19,920	29.96
Region						
North	922	27.69	14,485	22.937	15,407	23.17
Northeast	1,674	50.27	21,426	33.928	23,100	34.75
Southeast	384	11.53	9,656	15.29	10,040	15.1
South	124	3.72	7,697	12.188	7,821	11.76
Midwest	226	6.79	9,888	15.657	10,114	15.21
Total	3,330	100	63,152	100	66,482	100

**Table 2:** Prevalence and prevalence ratio (PR) * indicators of chronic noncommunicable diseases (CNCDs) in women receiving and not receiving the Bolsa Familia Program, System Risk and Protective Factors Surveillance for Chronic Diseases Telephone Survey (Vigitel), Brazil, 2016–2017.

Indicators	Bolsa família						PR_gross_ (A/B)	95%CI		PR_adjusted_ (A/B)	95%CI	
	Has (A)			Doesn’t have (B)								
	%	95%CI		%	95%CI							
Smoker	12.6	9.7	15.5	6.4	6.1	6.7	1.97	1.56	2.49	1.98	1.61	2.43
Overweight	57.98	54.02	61.95	49.22	48.65	49.80	1.18	1.10	1.26	1.21	1.14	1.29
Obesity	28.41	24.31	32.50	18.01	17.58	18.44	1.58	1.36	1.83	1.63	1.44	1.85
Recommended FLV	18.48	15.54	21.41	29.78	29.26	30.30	0.62	0.53	0.73	0.63	0.54	0.73
Consumption of Soft drinks	21.50	17.78	25.21	11.68	11.26	12.09	1.84	1.54	2.20	1.68	1.43	1.98
Consumption of Beans	66.27	62.63	69.91	52.91	52.34	53.48	1.25	1.18	1.32	1.25	1.18	1.32
Leisure time PA (≥ 150 min)	23.08	19.80	26.35	33.11	32.57	33.64	0.70	0.60	0.80	0.65	0.57	0.75
PA at home	71.96	68.27	75.66	48.48	47.91	49.06	1.48	1.41	1.56	1.35	1.29	1.41
Habit of watching television (3 h/day)	29.18	25.05	33.31	23.62	23.15	24.10	1.24	1.07	1.42	1.37	1.21	1.55
Alcohol abuse	12.18	9.68	14.68	11.26	10.86	11.66	1.08	0.88	1.33	0.88	0.72	1.07
Bad health self-assessment	8.34	5.76	10.93	4.96	4.72	5.20	1.68	1.23	2.30	2.04	1.56	2.66
Women (50 to 69 years) mammography	68.87	62.34	75.40	79.99	79.30	80.68	0.86	0.78	0.95	0.86	0.79	0.94
Women (25 to 64 years) Pap smear	75.73	71.87	79.59	83.74	83.20	84.27	0.90	0.86	0.95	0.91	0.87	0.95
Hypertension	25.58	21.81	29.35	26.76	26.30	27.22	0.96	0.82	1.11	1.46	1.29	1.64
Diabetes	8.43	6.13	10.74	8.73	8.45	9.00	0.97	0.73	1.27	1.66	1.33	2.07

* Age-adjusted; 95% CI: 95% confidence interval; FLV: fruits and vegetables; PA: physical activity.

**Table 3:** Prevalence and prevalence ratio (PR) * of indicators of chronic noncommunicable diseases (CNCDs) in women receiving and not receiving Bolsa Familia Program, with schooling (zero to eight years), System Risk and Protective Factors Surveillance for Diseases Chronicles by Telephone Survey (Vigitel), Brazil, 2016–2017.

Indicators	Bolsa família						PR_gross_(A/B)	95%CI		PR_adjusted_ (A/B)	95%CI	
	Have (A)			Doesn’t have (B)								
	%	95%CI		%	95%CI							
Smoker	15.81	11.05	20.57	8.73	8.06	9.40	1.81	1.33	2.47	1.51	1.16	1.97
Overweight	62.22	56.26	68.18	61.87	60.82	62.92	1.01	0.91	1.11	1.04	0.96	1.12
Obesity	33.68	27.23	40.13	26.56	25.63	27.50	1.27	1.04	1.54	1.24	1.06	1.45
Recommended FLV	15.77	11.53	20.00	24.23	23.31	25.15	0.65	0.50	0.85	0.71	0.56	0.89
Consumption of Soft drinks	16.72	11.38	22.07	10.22	9.45	11.00	1.64	1.18	2.27	1.12	0.83	1.50
Consumption of Beans	68.35	62.74	73.97	59.87	58.82	60.91	1.14	1.05	1.24	1.09	1.01	1.17
Leisure time PA (≥ 150 min)	18.63	13.70	23.57	22.19	21.30	23.08	0.84	0.64	1.10	0.79	0.63	0.98
PA at home	75.92	70.32	81.53	58.59	57.56	59.62	1.30	1.20	1.40	1.03	0.97	1.09
Habit of watching television (3 h/day)	30.79	24.33	37.25	28.41	27.46	29.36	1.08	0.88	1.34	1.11	0.94	1.31
Alcohol abuse	11.36	7.49	15.22	5.64	5.04	6.23	2.01	1.41	2.88	1.05	0.76	1.46
Bad health self-assessment	10.35	6.16	14.54	8.93	8.34	9.51	1.16	0.77	1.75	1.46	1.07	1.97
Women (50 to 69 years) mammography	67.61	59.86	75.36	73.55	72.32	74.78	0.92	0.82	1.03	0.92	0.82	1.03
Women (25 to 64 years) Pap smear	74.93	69.16	80.69	79.40	78.11	80.69	0.94	0.87	1.02	0.96	0.90	1.03
Hypertension	33.17	27.08	39.25	49.67	48.60	50.75	0.67	0.56	0.80	1.12	1.00	1.26
Diabetes	10.68	6.80	14.57	17.96	17.24	18.68	0.59	0.41	0.86	1.23	0.96	1.59

** Age-adjusted; 95% CI: 95% confidence interval; FLV: fruits and vegetables; PA: physical activity.

**Table 4:** Prevalence and prevalence ratio (PR) * indicators of chronic noncommunicable diseases (CNCDs) in women receiving and not receiving Bolsa Familia Program with schooling (nine to 11 years), System Risk and Protective Factors Surveillance for Diseases Chronicles by Telephone Survey (Vigitel), Brazil, 2016–2017.

Indicators	Bolsa família						PR_gross_(A/B)	95%CI		PR_adjusted_ (A/B)	95%CI	
	Have (A)			Doesn’t have (B)								
	%	95%CI		%	95%CI							
Smoker	8.83	6.18	11.47	6.39	5.85	6.94	1.38	1.01	1.89	1.54	1.14	2.09
Overweight	53.56	48.43	58.68	49.16	48.16	50.15	1.09	0.99	1.20	1.11	1.02	1.21
Obesity	22.60	18.23	26.97	17.55	16.81	18.28	1.29	1.06	1.57	1.29	1.08	1.54
Recommended FLV	19.16	15.22	23.11	27.74	26.86	28.62	0.69	0.56	0.85	0.81	0.67	0.97
Consumption of Soft drinks	28.19	22.79	33.60	13.60	12.82	14.38	2.07	1.70	2.53	1.79	1.50	2.14
Consumption of Beans	64.94	60.43	69.45	55.91	54.92	56.90	1.16	1.08	1.25	1.13	1.06	1.21
Leisure time PA (≥ 150 min)	26.53	22.38	30.67	32.04	31.11	32.97	0.83	0.71	0.97	0.82	0.71	0.95
PA at home	20.90	16.85	24.95	13.24	12.51	13.96	1.58	1.29	1.93	1.38	1.15	1.65
Habit of watching television (3 h/day)	71.05	66.41	75.68	56.24	55.24	57.24	1.26	1.18	1.35	1.18	1.11	1.25
Alcohol abuse	13.30	10.26	16.33	11.43	10.73	12.13	1.16	0.92	1.47	1.02	0.81	1.29
Bad health self-assessment	6.50	3.68	9.32	4.36	3.96	4.76	1.49	0.96	2.32	1.69	1.16	2.44
Women (50 to 69 years) mammography	75.86	66.50	85.23	81.99	80.91	83.07	0.93	0.82	1.05	0.92	0.82	1.04
Women (25 to 64 years) Pap smear	76.00	70.88	81.12	83.09	82.20	83.99	0.91	0.85	0.98	0.95	0.89	1.01
Hypertension	16.50	13.02	19.97	21.89	21.16	22.62	0.75	0.61	0.93	1.16	0.98	1.37
Diabetes	5.79	4.00	7.58	6.32	5.93	6.72	0.92	0.67	1.25	1.60	1.22	2.10

* Age-adjusted; 95% CI: 95% confidence interval; FLV: fruits and vegetables; PA: physical activity.

**Table 5:** Prevalence and prevalence ratio (PR) * indicators of chronic noncommunicable diseases (CNCDs) in women receiving and not receiving Bolsa Familia Program according to education (12 years or more), System Risk and Protective Factors Surveillance for Diseases Chronicles by Telephone Survey (Vigitel), Brazil, 2016–2017.

Indicators	Bolsa família						PR_gross_(A/B)	95%CI		PR_adjusted_ (A/B)	95%CI	
	Have (A)			Doesn’t have (B)								
	%	95%CI		%	95%CI							
Smoker	7.37	0.44	14.30	4.64	4.23	5.05	1.59	0.62	4.08	1.80	0.73	4.44
Overweight	47.36	36.21	58.51	39.67	38.78	40.56	1.19	0.94	1.51	1.27	1.01	1.61
Obesity	16.87	8.60	25.13	11.92	11.35	12.48	1.42	0.86	2.32	1.51	0.94	2.44
Recommended FLV	37.22	25.86	48.58	35.83	34.96	36.70	1.04	0.76	1.41	1.12	0.83	1.50
Consumption of Soft drinks	23.90	13.11	34.70	11.06	10.44	11.68	2.16	1.37	3.41	2.01	1.30	3.12
Consumption of Beans	56.30	45.65	66.95	44.92	44.01	45.84	1.25	1.04	1.52	1.23	1.02	1.47
Leisure time PA (≥ 150 min)	40.87	29.77	51.97	42.36	41.45	43.27	0.96	0.73	1.27	0.94	0.72	1.23
PA at home	44.10	33.42	54.78	33.85	32.99	34.71	1.30	1.02	1.66	1.25	0.99	1.58
Habit of watching television (3 h/day)	22.10	13.02	31.17	17.09	16.44	17.75	1.29	0.86	1.95	1.43	0.96	2.14
Alcohol abuse	12.78	4.64	20.91	15.39	14.67	16.10	0.83	0.44	1.57	0.73	0.39	1.39
Bad health self-assessment	1.86	0.32	3.41	2.49	2.19	2.78	0.75	0.32	1.73	0.77	0.32	1.83
Women (50 to 69 years) mammography	63.90	42.73	85.08	88.89	87.98	89.80	0.72	0.52	1.00	0.72	0.52	1.00
Women (25 to 64 years) Pap smear	81.41	72.29	90.54	86.92	86.22	87.63	0.94	0.84	1.05	0.96	0.86	1.07
Hypertension	13.07	6.78	19.36	13.70	13.18	14.23	0.95	0.59	1.55	1.67	1.07	2.60
Diabetes	4.40	-0.10	8.91	3.86	3.56	4.15	1.14	0.41	3.18	1.99	0.77	5.12

* Age-adjusted; 95% CI: 95% confidence interval; FLV: fruits and vegetables; PA: physical activity.
